# Ototoxicity of acetic acid on the guinea pig cochlea

**DOI:** 10.1186/s40463-015-0110-6

**Published:** 2015-12-14

**Authors:** Takafumi Yamano, Hitomi Higuchi, Takashi Nakagawa, Tetsuo Morizono

**Affiliations:** Section of Otorhinolaryngology, Department of Medicine, Fukuoka Dental College, 2-15-1 Tamura,Sawara-ku, Fukuoka, 814-0193 Japan; Department of Otorhinolaryngology, Fukuoka University School of Medicine, Jounan-ku Nanakuma 7-45-1, Fukuoka, 814-0180 Japan; Nishi Fukuoka Hospital, Nishi-ku Ikino-matsubara 3-18-8, Fukuoka, 819-8555 Japan

## Abstract

**Background:**

To evaluate the ototoxicity of acetic acid solutions.

**Methods:**

Compound action potentials (CAPs) of the eighth nerve were measured in guinea pigs before and after the application of acetic acid in the middle ear cavity. The pH values of the acetic acid solutions were pH 3.0, 4.0, and 5.0, and the application times were 30 min, 24 h, and 1 week.

**Results:**

Acetic acid solution (pH 3.0, *N* = 3) for 30 min caused no significant elevation in CAP threshold at 4 kHz, but a significant elevation in the threshold was noted for 8 kHz and clicks. Acetic acid solutions (pH 4.0 *N* = 6, 5.0 *N* = 5) for 30 min caused no significant elevation in CAP. Acetic acid solution (pH 4.0) for 24 h (*N* = 5) caused significant elevations of the CAP threshold for 8 kHz, 4 kHz, and for clicks. Acetic acid (pH 5.0) for 24 h (*N* = 3) caused a significant elevation of the CAP threshold for 4 kHz, but not for 8 kHz or clicks. Acetic acid (pH 5.0) for 1 week (*N* = 3) caused a small but significant elevation CAP the threshold for 8 kHz and 4 kHz tone bursts, but no significant change was noted for clicks.

**Conclusions:**

We found a significant toxic effect of acetic acid in guinea pigs on eighth-nerve compound action potentials when the pH was 5.0 or lower. Clearly, the stronger the acidity, and longer the exposure time, the more the CAP threshold was elevated.

## Background

The purpose of this study is to elucidate the effect of various acidity of acetic acid on the guinea pig cochlea. Ototoxicity of Burow’s solution on the guinea pig cochlea was reported by us (1). Main ingredient of Burow’s solution is 13 % aluminum acetate. Original Burow’s solution has a pH 3.5 which caused a significant reduction of compound action potential (CAP) when applied in the middle ear cavity for 30 min, while a two fold diluted Burow’s solution (pH 4.4) caused no reduction in CAP threshold. No study has been performed to determine ototoxicity of acetic acid with various pH.

## Methods

This protocol was approved by Fukuoka University Animal Ethics Committee.

### Animals

To evaluate the ototoxicity of various acidity (pH) of acetic acid, alubino Hartley guinea pigs of both genders (in total *N* = 30) were used. Animals selected had an average body weight between 300 and 400 g and had a positive Prayer reflex.

### Acetic acid

The test solution was freshly prepared by the Pharmacy Department of our University Hospital, and the pH was measured and adjusted to 3.0, 4.0, and 5.0, before each experiment. Osmotic pressure of acetic acid studied was 300 mOsm, the molecular weight was 60.

### Surgical procedure

The animals were anesthetized with sodium pentobarbital (30 mg/kg), and were secured in a custom-made head holder. Xylocaine (Astra Zeneca PLC, Osaka, Japan) 0.5 % was infiltrated into the surgical area before making the skin incision for access to the middle ear cavity. The tympanic bulla was exposed using a retro-auricular incision. A small hole, about 2 mm in diameter, was made using a dental drill, and the round window membrane was visualized with a 40× operating microscope.

### Sound system

Asynchronous tone bursts of 4 kHz, 8 kHz (1-ms rise and fall time, 10- ms plateau time), and click sounds were given as stimuli at a pulse rate of 20 per second, from 80 dB (re 20μPa) to thresholds with 10 dB decrements. The speaker used was a Telephonics TDH-39P, and the sound source was placed 10 cm away from the auricle. The free field sound pressure was monitored and calibrated with a Brüel & Kjær half-inch condenser microphone.

### Recording system and CAP measurement

An 0.08-mm-diameter Teflon-insulated silver wire with an exposed ball tip was carefully placed with a micromanipulator on the peripheral round window membrane. An Ag-AgCl reference electrode was placed in the neck muscles. The obtained CAP responses were averaged 200 times with a Traveler Express ER-22 (Biologic Systems Corp. USA.)

### Application of acetic acid

After the initial CAP was measured, the middle ear cavity was filled with acetic acid of various acidity. The amount of fluid necessary to fill the middle ear cavity was about 0.2 mls. Prior to CAP measurement, middle ear cavity of each animal was thoroughly dried using a tissue paper wick. We evaluated the following: (1–3) the effect on the action potential threshold of acetic acid (pH 3.0, 4.0, and 5.0) at 30 min after topical application, (4-5) the effect of 24 h application of acetic acid (pH 4.0, 5.0), (6) the effect of 1 week application of acetic acid (pH5.0).

### Analysis of the data

A threshold response was defined as an N1-P1 signal with amplitude of 10 μV. The change in the sound pressure level in decibels before and after drug application was defined as a change in hearing. The threshold change before and after drug application was compared, and a paired *t*-test was used to define statistical significance.

### Bacteriology

The bacteriostatic activity of acetic acid was studied using a disk diffusion assay. Bacteria obtained from patients of our clinic was two stocks of MRSA, which were diluted to 10^6^ FCU/ml, and cultured on an agar plate for 24 h. Acetic acid with pH 3.0, 4.0, 5.0, either 50 μl or 75 μl, was dropped on an 8 mm diameter disk, and placed on the agar plate. At another 24 h, the diameter of zones of inhibition of bacterial growth on the agar plate was measured.

## Results

**Fig. 1:**
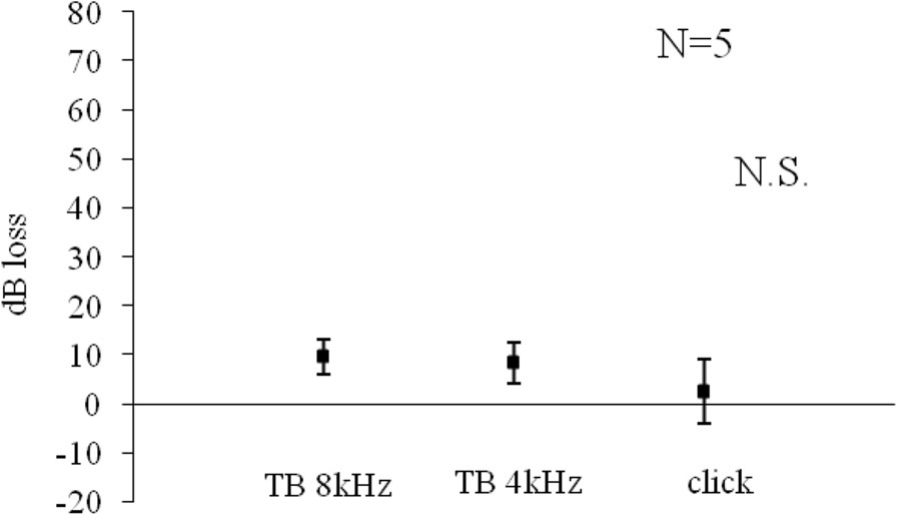
Saline for 30 min

Figure 1 shows changes in CAP threshold in decibel loss before and 30 min after saline control. No significant reduction in CAP was observed brfore and after saline application.

**Fig. 2:**
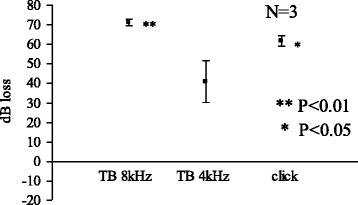
Acetic acid (pH 3.0) 30 min

Figure 2 shows changes in CAP threshold in decibel loss from control at 30 min. For 4 kHz, no significant elevation in CAP threshold was noted. For click stimulation and for 8 kHz, a significant elevation of the threshold was noted.

**Fig. 3:**
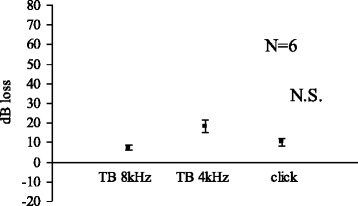
Acetic acid (pH 4.0) 30 min

**Fig. 4:**
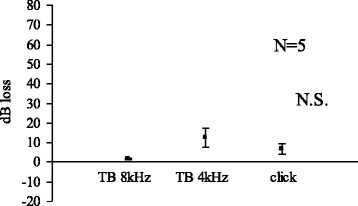
Acetic acid (pH 5.0) 30 min

Figure 3 shows changes in CAP with acetic acid pH 4.0 at 30 min. No significant elevation in CAP threshold was noted at 30 min. Significant difference exists between pH 3.0 and pH 4.0 solution at 30 min. Figure 4 shows the results from acetic acid pH 5.0 at 30 min. No change in CAP threshold was noted at 30 min.

**Fig. 5:**
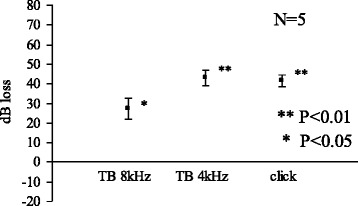
Acetic acid (pH 4.0) 24 h

At 24 h, acetic acid (pH 4.0) caused significant elevation of CAP threshold for 8 kHz, 4 kHz, and for click sounds (Fig. 5). Compared with the results at 30 min, ototoxicity became evident at 24 h.

**Fig. 6:**
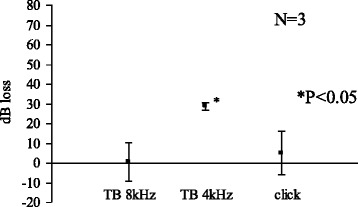
Acetic acid (pH 5.0) 24 h

At 24 h, acetic acid (pH 5.0) caused a significant elevation of CAP threshold with tone burst of 4 kHz, but no significant elevation for 8 kHz or for click sounds (Fig. 6).

**Fig. 7:**
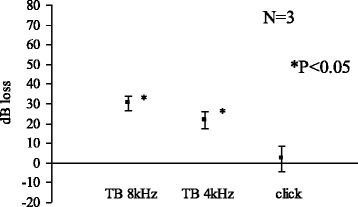
Acetic acid (pH 5.0) 1 week

At 1 week, acetic acid (pH 5.0) caused small but a significant elevation in CAP threshold for 8 kHz and 4 kHz tone burst. No significant change was noted for click sound (Fig. 7).

### Bacteriology

The diameter of the zone of inhibition for two stocks of *MRSA* is shown in Table 1. The zone of inhibition in mm is shown. The larger zones of inhibition indicate better antimicrobial effect. Either 50 μl or 75 μl, was used. The diameter of the disk was 8 mm. At 24 h, Bacteriostatic activity was noted for pH 3.0, 4.0, and 5.0. The bacteriostatic activity was concentration dependent. A larger amount of acetic acid caused more bacteriostatic activity.

**Table 1:** Bacteriostatic activity for either 50 or 75 μl of acetic acid

		pH3.0	pH4.0	pH5.0
MRSA1	50 μL	35	32	21
	75 μL	39	33	26
MRSA2	50 μL	37	30	23
	75 μL	40	35	26
				(mm)

## Discussion

The bactericidal effect of acetic acid has been well known empirically. Recently, Burrow’s solution has gained popularity as effective solution in treating intractable ear discharge with fungi and with methicillin-resistant *staphylococcus aureus* (MRSA). The primary ingredient of this solution is acetic acid and 13 % aluminum acetate. In our previous study [1], use of original Burrow’s solution (pH3.5) for 30 min caused a significant elevation of CAP threshold, a two-fold diluted Burrow’s solution (pH 4.4) for 30 min caused no change in CAP threshold [1].

VoSol otic solution (ECR Pharmaceuticals Co. Inc) is a commonly available ear drops in the USA, which contains 2 % acetic acid in a propylene glycol vehicle containing propylene glycol diacetate (3 %), benzentonoum chloride (0.02 %), and sodium acetate (0.015 %). This otic solution is buffered at pH3.0 for use in the external canal. Adverse effect of VoSol solution has been reported previously [2–4]. Application of 2 % acetic acid or VoSol on the round window membrane caused a reduction of pH in the perilymph, in the endolymph, and a reduction of endocochlear DC potentials (EP). The reduction started only a few minutes after the application. They concluded that otic preparation containing acetic acid penetrates the round window membrane within a few minutes, and causes an inhibition of Na+, K-ATPase activity of the stria vascularis. The effect was much stronger for the VoSol solution than for acetic acid, probably due to synergistic effects of acetic acid and propylene glycol. Propylene glycol causes damage of the round window membrane, thus the diffusion of acetic acid becomes greater.

Thorp et al [5–7] reported antibacterial activity of acetic acid and Burow’s solution in vitro and also in vivo. However, otototoxic effect of this solution was not addressed. No systemic study has been performed to determine the ototoxicity of acetic acid alone.

We varied the pH of acetic acid to 3.0, 4.0, and 5.0. Also we varied the duration of acetic acid in the middle ear cavity for 30 min, 24 h, and 1 week.

We found a significant toxic effect of acetic acid in guinea pigs on eighth nerve compound action potentials (CAP).

Clearly, stronger the acidity, more the elevation of CAP threshold, and longer the exposure time, more the elevation of CAP threshold.

We limited the use of animals as small as possible yet to obtain significant results. In the Figs. 1, 2, 3 and 4, standard errors shown as a vertical line indicated that the variability of the change among animals in each groups is small.

In the group of Acetic acid (pH5.0) for 1 week, probably most of the acetic acid leaked out from Eustachian tube at the time we took the measurement. Also, tissue fluids from the middle ear mucosa would dilute the acetic acid. Comparison of the group of acetic acid (pH 5.0) for 30 min, 24 h and 1 week revealed that longer exposure time is more harmful to the cochlea, suggesting the toxic effect of the acetic acid is not reversible.

It is impossible to completely exclude the possibility of a conductive hearing loss caused by the fluid in the tympanic bulla. However, we believe conductive component of the hearing loss is minimum after drying the middle ear cavity with wicks of tissue paper.

Although acidic solutions have been used in the middle ear cavity as ear drops or irrigation, ototoxicity of the acidic solution has not been addressed adequately. Our previous study [1] showed Burrow’s solution with pH 3.5 for 30 min caused a significant reduction in CAP, yet a two-fold dilute Burrow’s solution (pH 4.4) for 30 min caused no reduction in CAP. Our current experiment suggests ototoxicity becomes evident at pH 3.0 at 30 min, which is in good agreement with the previous study.

In the clinical settings, it is advisable to avoid allowing the solution to contact the round window membrane for extended times.

## Conclusions

No systemic study has been performed to determine the ototoxicity of acetic acid alone. We varied the pH of acetic acid to 3.0, 4.0, and 5.0. Also we varied the duration of acetic acid in the middle ear cavity for 30 min, 24 h, and 1 week.

We found a significant toxic effect of acetic acid in guinea pigs on eighth nerve compound action potentials (CAP) when the pH is 5.0 or less. Clearly, stronger the acidity, more the elevation of CAP threshold, and longer the exposure time, more the elevation of CAP threshold.
